# Rectal ectopic pregnancy diagnosed through laparoscopy and pathological analysis: An extremely rare case report

**DOI:** 10.1097/MD.0000000000043344

**Published:** 2025-07-11

**Authors:** Cheng Li, Huixing Li, Yanli Feng

**Affiliations:** a Department of Gynecology, Jining Medical University, Jining City, Shandong Province, China; b Department of Gynecology, Affiliated Hospital of Jining Medical University, Jining City, Shandong Province, China.

**Keywords:** case report, laparoscopic, pathological diagnosis, rectal ectopic pregnancy

## Abstract

**Rationale:**

Rectal ectopic pregnancy (EP) is an extremely rare subtype of abdominal pregnancy, posing significant diagnostic challenges due to its infrequent occurrence, atypical clinical manifestations, and imaging findings that mimic other medical conditions. Delayed diagnosis may lead to life-threatening complications such as intra-abdominal hemorrhage. This case highlights the importance of comprehensive laparoscopic exploration and pathological confirmation in identifying rare ectopic implantation sites.

**Patient concerns:**

A 40-year-old female presented with a 1-day history of abdominal pain, 8-day vaginal spotting, and 39-day amenorrhea. Gynecological ultrasound revealed a mixed echogenic area posterior to the uterus, endometrial hyperechoic area, and bilateral fallopian tube thickening, with serum beta-human chorionic gonadotropin levels at 969.19 IU/L.

**Diagnoses:**

Preoperative suspicion of EP was confirmed intraoperatively via laparoscopic exploration, which revealed a 0.5 cm rectal wall defect in the rectovaginal pouch with blood clots. Pathological analysis of pelvic clots identified chorionic villi, confirming rectal EP.

**Interventions:**

Laparoscopic management included clot evacuation, electrocoagulation of the rectal defect, suture hemostasis, and postoperative oral mifepristone (50 mg bid) to prevent residual trophoblastic tissue.

**Outcomes:**

Serum beta-human chorionic gonadotropin levels declined to negative within 3 weeks postoperatively, with no evidence of complications during follow-up.

**Lessons:**

This case emphasizes the necessity of thorough laparoscopic examination of the pelvic and abdominal cavities in suspected EP to avoid missed diagnoses in concealed implantation sites, such as the rectal wall.

## 1. Introduction

The implantation of a fertilized ovum outside the uterine cavity is termed ectopic pregnancy (EP), often referred to as extrauterine pregnancy.^[1]^ Ectopic pregnancies predominantly occur in the fallopian tubes, accounting for 95% of cases, while rarer instances include ovarian, abdominal, cervical, and broad ligament pregnancies.^[2]^ Abdominal EP is recognized as one of the rarest forms of ectopic gestation, with an indeterminate incidence rate and mortality estimates ranging from 1 in 10 to 1 in 200,^[3]^ with assisted reproductive technologies identified as a significant risk factor.^[4]^ This report details an exceptionally rare case of abdominal EP affixed to the anterior rectal wall in a spontaneous conception. The article aims to provoke thought among clinicians regarding the diagnosis and management of abdominal pregnancies.

## 2. Case presentation

Ethical approval was formally waived by the Medical Ethics Committee of Jining Medical University in accordance with Article 22 of its charter (JY Hospital Document No. 89, 2023), which exempts retrospective case reports using completely anonymized clinical data without therapeutic intervention. Written informed consent was obtained from the patient for publication.

A 40-year-old female patient, with a history of 2 vaginal deliveries and 1 cesarean section, presented to the outpatient department with primary complaints of amenorrhea for 39 days, vaginal spotting for 8 days, and abdominal pain for 1 day as of September 2, 2024. She reported no surgical or allergic history, with her last menstrual period dated July 25, 2024. The patient was alert and hemodynamically stable, with a blood pressure of 126/76 mm Hg and a pulse rate of 84 beats/min.

**Figure 1.**
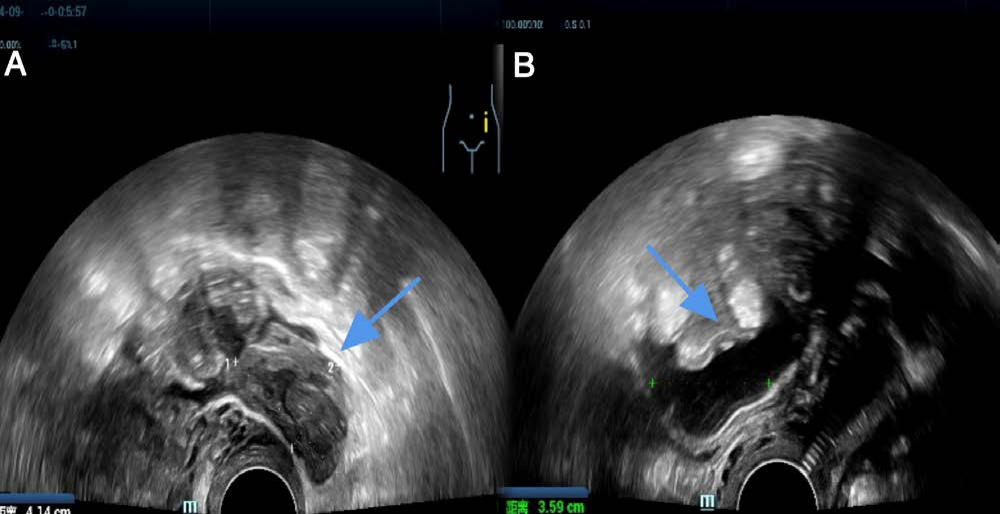
Preoperative transvaginal ultrasound findings. (A) Sagittal view showing a mixed echogenic mass (arrowheads) posterior to the uterine body, composed of heterogeneous solid and cystic components. (B) Transverse view demonstrating an anechoic fluid collection (arrowheads) surrounding the uterus, indicative of intraperitoneal bleeding.

Gynecological examination revealed thickening and marked tenderness in the right adnexal region, with no other significant findings. Laboratory tests indicated a blood beta-human chorionic gonadotropin (β-hCG) level of 969.19 IU/L, exceeding the normal range for nonpregnant women, which is <5 IU/L. Transvaginal ultrasound demonstrated a large uterus measuring 58 mm × 58 mm × 51 mm, with normal morphology and an endometrial thickness of approximately 1.2 cm. A hyperechoic area measuring approximately 8 mm × 5 mm × 6 mm was noted, characterized by well-defined borders and heterogeneous internal echogenicity. A mixed echogenic area posterior to the uterine body measured approximately 41 mm × 25 mm × 40 mm (Fig. 1A), with clear borders and poor translucency in the cystic portion. The solid component exhibited uneven echogenicity, with a small blood flow signal detected at the periphery. Bilateral tubal thickening was observed, measuring approximately 1.1 cm on the right and 0.9 cm on the left, with the mixed echogenic areas appearing to connect to the right adnexa. An anechoic area surrounding the uterus measured approximately 3.6 cm in depth (Fig. 1B), with acceptable sound transmission. The patient was subsequently admitted to the hospital with a provisional diagnosis of EP.

**Figure 2.**
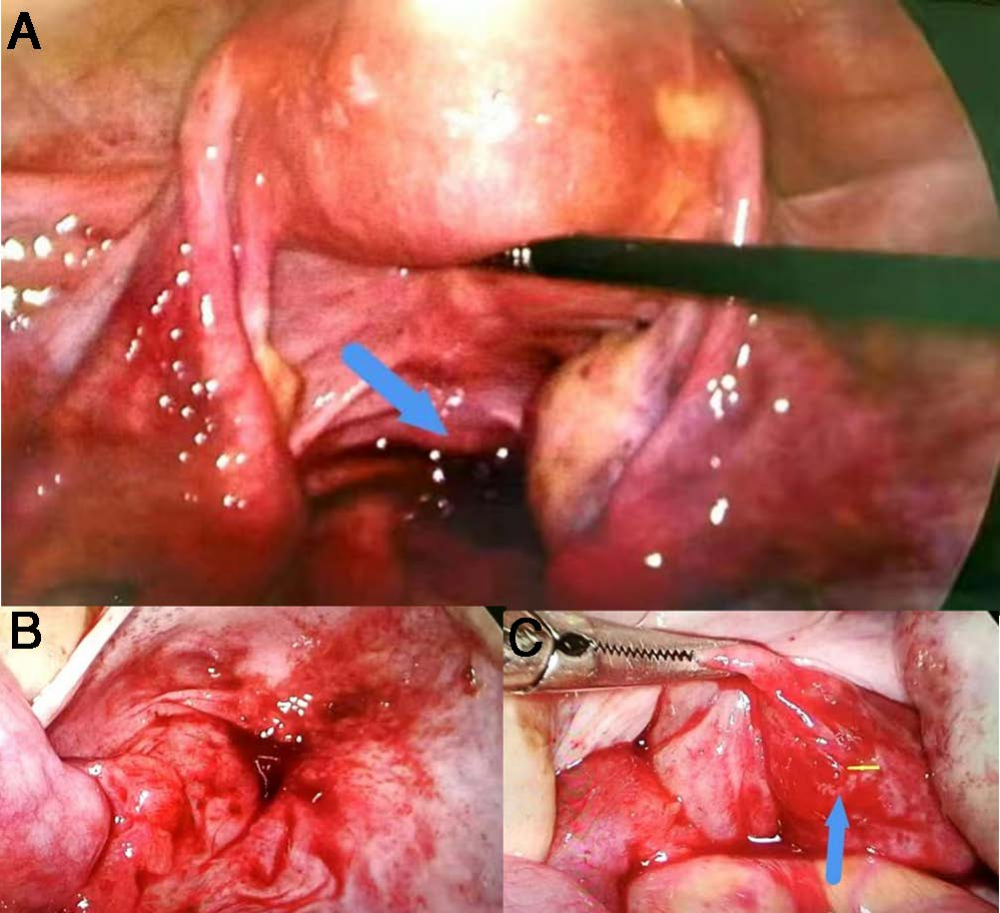
Intraoperatively laparoscopic examination (A) indicates an accumulation of blood in the rectouterine trap, with bilateral tubes intact without rupture orifices, (B) removal of blood clots did not reveal any obvious lesions, (C) careful exploration reveals a rupture in the rectal surface.

**Figure 3.**
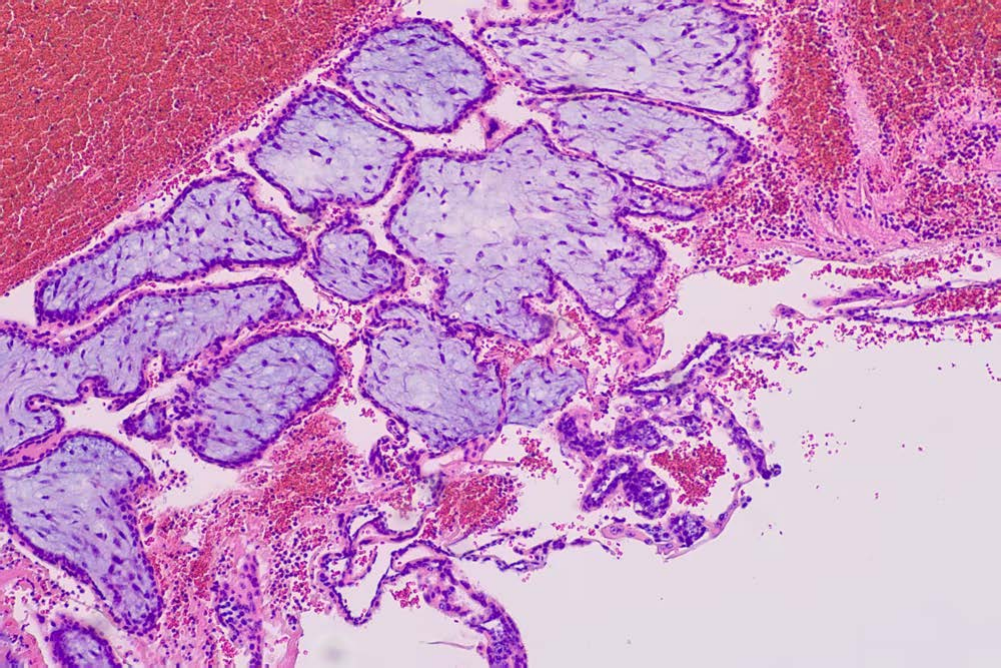
Pathological examination of pelvic blood clots (H&E staining) revealed chorionic villi tissues. H&E = hematoxylin-eosin staining.

The preoperative assessment of the patient was thorough, revealing no contraindications for surgical intervention. Given the clinical, laboratory, and imaging results, alongside the patient’s significant lower abdominal pain and the potential for a ruptured EP, it was determined that surgery should be conducted promptly, 2 hours post-admission. Intraoperatively (Fig. 2), approximately 600 mL of stagnant pelvic blood was observed, accompanied by numerous dark blood clots, particularly within the rectouterine pouch. The uterus appeared normal in size and morphology, while the bilateral fallopian tubes exhibited slight thickening without any overt abnormalities or ruptures on their surfaces. Upon irrigation, a 5 mm diameter defect was identified on the intestinal wall at the rectouterine pouch, with a small amount of fresh blood oozing from the site. The defect was addressed through unipolar bulge electrocoagulation of the bowel wall trauma, followed by the application of 3/0 absorbable sutures to halt the bleeding. Subsequent to this, a warm saline rinse was administered to the pelvic cavity, and no further bleeding was detected laparoscopically. Hysteroscopic evaluation revealed no gestational sac within the uterine cavity. The intraoperative diagnosis was “abdominal pregnancy?”. Pathological examination confirmed the presence of chorionic villi within the pelvic blood clot (Fig. 3), substantiating the diagnosis of abdominal pregnancy. Postoperatively, the patient received 50 mg of oral mifepristone twice daily for treatment, with hCG levels monitored regularly. Serum β-hCG levels decreased from 969.19 IU/L preoperatively to 608.05 IU/L (POD1) and 296.03 IU/L (POD3). Due to the rare location of the implant, close monitoring was required for potential postoperative complications, including healing of the rectal wall, active bleeding, and drug reactions. The patient achieved complete recovery and was discharged from the hospital 3 days after surgery. One week after the operation, the patient was reviewed in the outpatient clinic. Serum β-hCG level had decreased to 36.80 IU/L (POD7) and achieving undetectable levels at 3 weeks. Transvaginal ultrasound at 1-week follow-up showed complete resolution of pelvic fluid and adnexal masses.

## 3. Discussion

Abdominal pregnancy is classified as a distinct form of EP, which can be categorized into primary and secondary abdominal pregnancies. Primary abdominal pregnancy occurs due to the fimbriae’s failure to capture the ovulated follicle, whereas secondary abdominal pregnancy is thought to arise following the early rupture or abortion of a tubal pregnancy or uterine perforation of an intrauterine pregnancy into the peritoneal cavity. The criteria for primary peritoneal pregnancy, as delineated by Studdiford in 1942,^[5]^ include:

1. Presence of normal tubes and ovaries with no evidence of recent or past pregnancy.
2. No evidence of uteroplacental fistula.
3. The presence of a pregnancy related exclusively to the peritoneal surface and early enough to eliminate the possibility of secondary implantation after primary tubal abortion.

In the current case, the occurrence of rectal EP without predisposing factors such as a history of assisted reproductive technologies, familial predisposition, or tubal anomalies is exceedingly uncommon. Laparoscopic evaluation revealed that the uterus, fallopian tubes, and ovaries appeared normal, with no evidence of rupture. A meticulous examination ultimately identified a rupture on the rectal surface, which was the origin of pelvic hematoma. Subsequent pathological analysis indicated the presence of chorionic villi within the pelvic blood clot, fulfilling the diagnostic criteria for primary peritoneal pregnancy. The integration of laparoscopic and pathological findings confirmed the diagnosis of rectal pregnancy.

It is imperative to recognize that diagnosing rectal EP poses significant challenges. Firstly, while gynecological ultrasound remains the most effective modality for diagnosing gynecological conditions,^[6]^ it often fails to detect ectopic pregnancies located in atypical sites. Secondly, in cases where EP is suspected, particularly with severe abdominal pain or unstable vital signs, prompt surgical intervention is warranted to facilitate definitive diagnosis and management intraoperatively. Furthermore, during laparoscopic procedures, a comprehensive examination of the pelvis, abdominal cavity, and surrounding organs is essential, as ectopic pregnancies may implant in abdominal structures such as the liver,^[7]^ rectum, and others. In this instance, due to the patient’s persistent abdominal pain, laparoscopic exploration was conducted 2 hours post-admission, allowing for timely intervention that mitigated potential complications.

During the exploration, the uterus and adnexa appeared largely normal, with no initial rupture detected; however, a rupture on the rectal surface was identified following thorough investigation, underscoring the necessity for meticulous laparoscopic exploration. Postoperatively, mifepristone was administered to avert the risk of persistent EP due to retained trophoblastic tissue. The patient’s β-hCG levels demonstrated a consistent decline during postoperative follow-up, marking this case as a successful intervention with notable clinical implications.

This report has several limitations: the extreme rarity of rectal implantation limits generalizability; preoperative MRI could have better characterized the rectal lesion but was deferred due to hemodynamic urgency; single-center experience without long-term fertility follow-up.

## 4. Conclusion

This effective management of rectal pregnancy underscores the importance of prompt laparoscopic exploration in cases with ambiguous ultrasonographic findings, emphasizing the need for thorough and vigilant examination to prevent underdiagnosis and misdiagnosis, which could jeopardize patient safety.

## Author contributions

**Writing – original draft:** Cheng Li.

**Writing – review & editing:** Huixing Li, Yanli Feng.
